# Prussian Blue/Chitosan Micromotors with Intrinsic Enzyme-like Activity for (bio)-Sensing Assays

**DOI:** 10.1021/acs.analchem.1c05173

**Published:** 2022-04-01

**Authors:** Roberto María-Hormigos, Águeda Molinero-Fernández, Miguel Ángel López, Beatriz Jurado-Sánchez, Alberto Escarpa

**Affiliations:** † Department of Analytical Chemistry, Physical Chemistry and Chemical Engineering, University of Alcala, Alcala de Henares E-28871, Madrid, Spain; ‡ Chemical Research Institute ″Andrés M. del Río”, University of Alcala, Alcala de Henares E-28871, Madrid, Spain

## Abstract

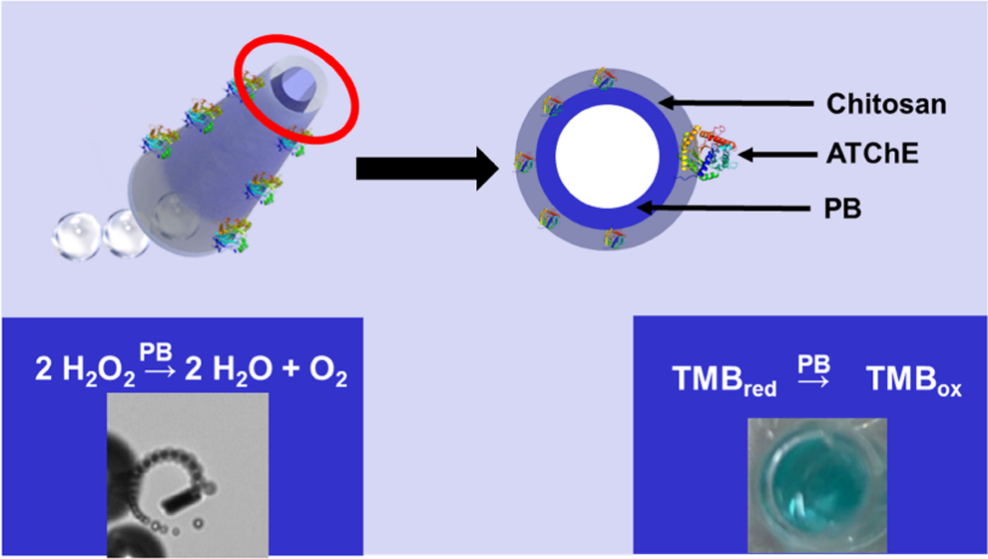

Prussian Blue (PB)/chitosan enzyme mimetic tubular micromotors are used here for *on-the-fly* (bio)-sensing assays. The micromotors are easily prepared by direct deposition of chitosan into the pores of a membrane template and in situ PB synthesis during hydrogel deposition. Under judicious pH control, PB micromotors display enzyme mimetic capabilities with three key functions *on board*: the autonomous oxygen bubble propulsion (with PB acting as a catalase mimic for hydrogen peroxide decomposition), 3,3′,5,5′-tetramethylbenzidine (TMB) oxidation (with PB acting as a peroxidase mimic for analyte detection), and as a magnetic material (to simplify the (bio)-sensing steps). In connection with chitosan capabilities, these unique enzyme mimetic micromotors are further functionalized with acetylthiocholinesterase enzyme (ATChE) to be explored in fast inhibition assays (20 min) for the colorimetric determination of the nerve agent neostigmine, with excellent analytical performance in terms of quantification limit (0.30 μM) and concentration linear range (up to 500 μM), without compromising efficient micromotor propulsion. The new concept illustrated holds considerable potential for a myriad of (bio)-sensing applications, including forensics, where this conceptual approach remains to be explored. Micromotor-based tests to be used in crime scenes are also envisioned due to the reliable neostigmine determination in unpretreated samples.

## Introduction

Artificial enzymes are nanomaterials with enzyme-like characteristics, which have been explored for more than 50 years as alternatives to natural enzymes due to their prolonged stability, low cost, and tunable activities.^1,2^ The mimetic capabilities of Prussian Blue (PB) are associated with its unique structure.^3^ Indeed, it is a mixed-valence compound with the general structure Fe_4_[Fe(CN)_6_]_3_ × *n*H_2_O with iron atoms in two different oxidation states and interconnected by C–N pairs. This results in the presence of different redox potentials in the same structure, making PB an efficient electron transporter with catalase and peroxidase-like activities.^3,4^ Self-propelled micromotors are micro- and nanodevices capable of autonomous motion in solution. Micromotors can be considered as another type of enzyme-like nanomaterials due to their capacity to decompose different fuels such as hydrogen peroxide in catalytic layers in a similar fashion as the catalase enzyme.^5−7^ A myriad of micromotors have been synthetized and functionalized in different shapes and with variable techniques, such as tubular micromotors by template electrosynthesis,^8^ Janus structures by physical vapor deposition,^9^ and bipolar electrochemistry^10^ or magnetic helices by laser writing.^11^ First designs integrate catalytic layers such as Pt for decomposition of hydrogen peroxide and oxygen bubble generation for efficient propulsion.^8^ The requirement of an expensive catalyst such as Pt and the low biocompatibility of peroxide fuel led to the exploration of alternative catalysts such as Mg as a reactive material,^12^ enzymes, or biohybrid propulsion modes.^13^ Fuel-free biocompatible designs such as magnetic-, light-, or ultrasound-propelled micromotors were also proposed as alternatives in the biomedical or environmental fields.^14^ The combination of enzymes with micromotors is a convenient alternative in which (bio)-propulsion represents a versatile and powerful alternative. From the first demonstration of the catalase/glucose oxidase combination into carbon nanotubes for autonomous propulsion in the presence of glucose,^15^ the field has rapidly evolved, demonstrating the convenience of the combination of urease, glucose oxidase, aldolase, etc., with different micromotor structures for propulsion in the presence of the corresponding substrates.^16−18^ This holds considerable promise for in vivo drug delivery^19^ or analytical sensing.^20,21^ Inspired by the previous enzyme-based micromotors and the enzyme-like behavior of PB, herein we report the synthesis of PB-based micromotors as artificial enzymes for (bio)-sensing assays. Neostigmine, a cholinesterase inhibitor drug that acts as a nerve agent, is used as the relevant analyte in forensic and other related fields. PB has been previously used in connection with Janus micromotors for buoyancy-induced displacement^22^ or as a template for the synthesis of iron oxide micromotors for water treatment.^23,24^ Yet, to the best of our knowledge, this is the first time that PB has been used in connection with micromotors for (bio)-sensing approaches. In the following sections, we will illustrate first the synthesis and propulsion of micromotors as well as the multienzyme mimetic features. Next, the micromotors will be used in colorimetric assays in connection with 3,3′,5,5′-tetramethylbenzidine (TMB) for nerve agent determination, using neostigmine as the target analyte. To this end, PB-based micromotors are functionalized with the enzyme acetylthiocholinesterase (ATChE), which is inhibited by neostigmine. Such inhibition hampers the conversion of ATCh into TCh. Thus, the poisoning of PB by TCh is prevented, acting as an active peroxidase mimic and promoting the oxidation of reduced TMB (TMB_red_) to its blue form (TMB_ox_). Thus, a higher concentration of neostigmine will produce a higher colorimetric signal. This principle of detection can be extended to a myriad of analytes that act as ATChE inhibitors, such as nerve agents or pesticides.

## Experimental Section

### Materials and Methods

Chlorohydric acid (cat. 320331), iron(III) chloride (cat. 8.03945), chitosan (cat. 448869), acetylcholine chloride (cat. A6625), TMB (cat. 860336), neostigmine bromide (cat. N2001), sodium cholate (cat. C9282), acetic acid (cat. 1.00063), glutaraldehyde solution 25% in water (cat. 354400), and acetylcholinesterase from *Electrophorus Electricus* (electric eel), Type V-S, lyophilized powder, 1527 units/mg protein (cat. C2888) were purchased from Sigma-Aldrich. Potassium hexacyanoferrate(III) (cat. 131503), ethanol (cat. 141086), and methanol (cat. 321091) were purchased from Panreac. Hydrogen peroxide (cat. 231-765-0) was purchased from Labbox. Coffee, tea, beer, and chamomile herbs were obtained from the local market. Beer was sonicated for 10 min for degasification. Coffee was prepared from its capsule in a commercial coffee machine with 100 mL of tap water. Tea and chamomile herbs were prepared in 250 mL of boiling tap water.

Scanning electron microscopy (SEM) and energy-dispersive X-ray (EDX) images were obtained with a JEOL JSM 6335F instrument using an acceleration voltage of 10 kV. An inverted optical microscope (Nikon Eclipse Instrument Inc. Ti-S/L100), coupled with 20× and 40× objectives, and a Hamamatsu digital camera C111440 and NIS Elements AR 3.2 software, was used for capturing movies at the rate of 50 frames per second. Hydrogen peroxide solutions (3, 5, and 10%) were used as chemical fuels. UV–visible experiments were carried out using a Synergy HTX (Biotek) microplate reader, scanning from 500 to 850 nm and recording the signal at the maximum of TMB_ox_ at 650 nm. Magnetic susceptibility of PB/chitosan dry micromotors was obtained using a magnetic susceptibility balance Mk1. Balance read-out was corrected using sample mass and height, room temperature, and a HgCo(SCN)_4_ magnetic standard to obtain the final micromotors’ magnetic susceptibility.

### Prussian Blue/Chitosan Micromotor Synthesis

Chitosan solution was prepared by dissolving 0.33 g of chitosan in 50 mL of 0.1 M acetic acid under vigorous stirring. Then, 0.5 M K_3_[Fe(CN)_6_] and FeCl_3_ solutions were prepared by dissolving the salts in 20 mL of HCl/KCl 0.1 M solution. All solutions were stored in the fridge in the absence of light. PB/chitosan micromotors were prepared by in situ PB synthesis during chitosan deposition into the 5 μm diameter conical pores of a polycarbonate membrane (cat. 7060-2513; Whatman, New Jersey). First, the membrane was placed with the pores facing up in a Petri dish. Then, the precursors (chitosan, K_3_[Fe(CN)_6_], and FeCl_3_) were added simultaneously to obtain a 1:1:8 K_3_[Fe(CN)_6_]/FeCl_3_/chitosan Prussian brown solution. Subsequently, the Petri dish was placed in an ultrasound bath for 10 min to displace the gas in the membrane pores. After that, the Petri dish was heated at 50 °C for 2 h in an oven. This results in the formation of a viscous Prussian green/hydrogel mixture. After washing the membrane with ultrapure water, a 5% glutaraldehyde solution was added for 30 min to promote chitosan hydrogel cross-linking and PB generation. Next, the membrane was gently polished to eliminate the excess of hydrogel and the micromotors were released by sequential treatment with methylene chloride (30 min, 2 times), isopropanol, ethanol, and ultrapure water (18.2 Ω cm), with 3 min centrifugation following each wash. The template preparation method resulted in reproducible micromotors. Acetylcholinesterase lyophilized powder was reconstituted in water to obtain a solution of 1527 U/mL (1 mg/mL) of the enzyme. For modification, the micromotors were incubated in 50 μL of acetylcholinesterase solution (200 U/mL) at 37 °C for 4 h under stirring at 950 rpm. Then, the micromotors were washed twice with 0.1 M phosphate buffer at pH 8.0 and stored at 4 °C for further use within a day

### Colorimetric Assays

Inhibition experiments for neostigmine determination were carried out by adding the acetylcholinesterase functionalized micromotors into a 1.5 mL Eppendorf. Then, neostigmine solution in 0.1 M phosphate buffer pH 8.0 was added and incubated for 10 min at 37 °C under stirring at 950 rpm. After that, ATCh was added as the first substrate in 0.1 M phosphate buffer pH 8.0 and incubated for 5 min at 37 °C under stirring at 950 rpm. Then, TMB_red_ was added as a secondary substrate in 0.2 M acetate/acetic acid buffer at pH 4.0, along with NaCh in 30% H_2_O_2_ solution. Final concentrations during the assay were 1 00 000 micromotors/mL, 1 mg/mL ATCh, 0.1 mg/mL TMB, 1.5% NaCh, and 10% H_2_O_2_. In all colorimetric assays, 300 μL of the supernatant was collected and transferred to a 96-well plate 1 min after H_2_O_2_ addition. A magnet was used to trap the micromotors in the bottom of the Eppendorf to simplify bioassay steps and to avoid interference in the MTT measurements. In all cases, TMB_ox_ was monitored by scanning from 500 to 850 nm in a microplate reader. Assays in the absence of neostigmine were carried out without the neostigmine incubation step.

## Results and Discussion

### PB/Chitosan Micromotors: Conceptual Analytical Design for Enzyme Inhibition-Based Assays for Neostigmine Detection

**Figure 1:**
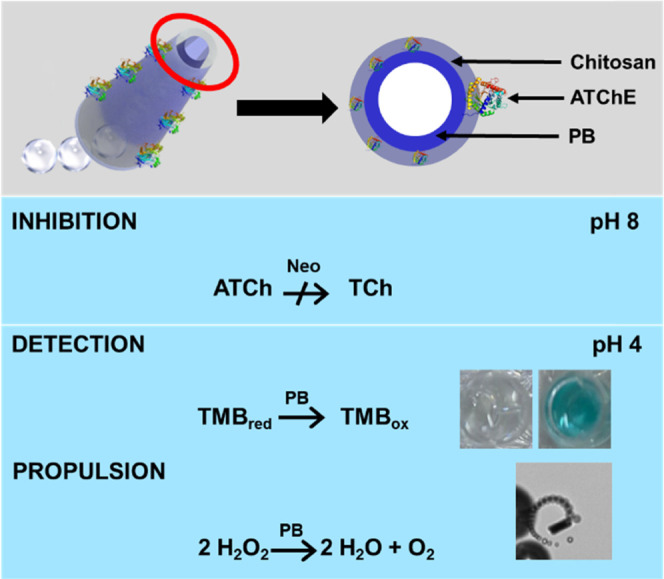
Schematic of the enzyme mimetic PB/chitosan/ACThE micromotors for neostigmine (Neo) biosensing.

Figure 1 shows a schematic of the neostigmine assay using PB/chitosan micromotors functionalized with the ATChE. The outer hydrogel layer was used for direct trapping/adsorption of ATChE for further neostigmine inhibition-based assay at pH 8. Then, in a second step, colorimetric neostigmine detection was carried out at pH 4. Interestingly, at pH 4, the PB inner layer exhibits enzymatic mimics, allowing the oxidation of TMB into a blue color in colorimetric assays (as peroxidase-like) without compromising efficient micromotor propulsion. The bioassay principle is based on the following reactions.

(1) In the absence of neostigmine:
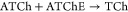

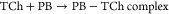

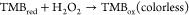

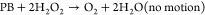
(2) In the presence of neostigmine:
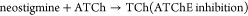

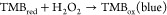

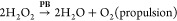

### PB/Chitosan Micromotor Synthesis and Characterization

**Figure 2:**
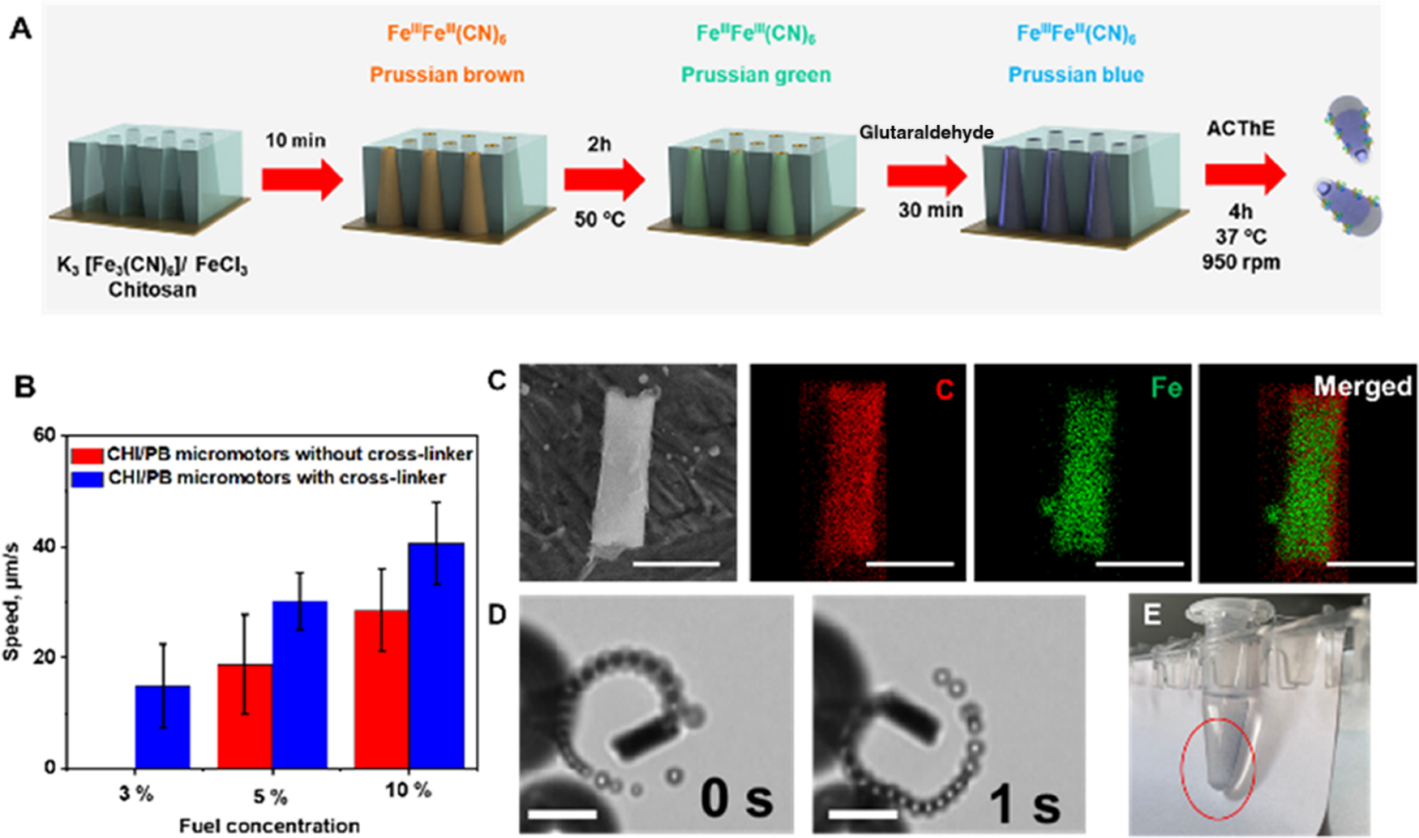
(A) Schematic of the template-synthesis of chitosan/PB/ATChE micromotors. (B) Effect of cross-linking with glutaraldehyde on the speed of micromotors. (C) SEM and EDX mapping illustrating the morphology and element distribution of micromotors. (D) Time-lapse microscopy images (taken from Video S1) showing the micromotor propulsion in 10% H_2_O_2_ and 1.5% Tween-20. (E) Magnetic properties of the micromotors. Scale bars are 10 μm (SEM images in C) and 20 μm (time-lapse images in D).

Figure 2 illustrates a schematic of the fabrication protocol of PB/chitosan micromotors and their functionalization with the ATChE enzyme. The PB-based micromotors were synthetized using a biocompatible hydrogel to impart the assay with biocompatibility features. As will be discussed, the hydrogel will also play a critical role in the stabilization and trapping of the PB layer. As can be seen in Figure 2A, micromotor synthesis was carried out in several steps. In a first step, the chitosan solution and the PB precursors are added to the 5 μm polycarbonate membrane template, followed by mixing in an ultrasound bath to eliminate the gas inside the pores of the membrane template and allow wettability of the inner space of the membrane. During this step, the precursor ions of PB get mixed and form the intermediate complex Prussian Brown, which is highly unstable.^26^ In a second step, the mixture is heated at 50 °C for 2 h, promoting chitosan deposition into the pores and allowing the generation of Prussian Green.^27^ In a third step, a glutaraldehyde solution is added to promote the cross-linking of the chitosan chain, thus conferring rigidity to the micromotors while trapping the as-generated Prussian Green particles inside of the hydrogel structure. In this sense, chitosan plays an important role during the PB micromotor synthesis as a matrix for solid encapsulation and entrapment, allowing for the fabrication of new catalytic systems using PB as a catalyst. Moreover, glutaraldehyde also acts as a mild reduction agent, which promotes the reduction of Prussian Green into the final PB. The generation of PB can be observed after 30 min of glutaraldehyde addition or after several hours if glutaraldehyde is not added to promote chitosan cross-linking.^28^ After membrane dissolution and micromotor removal, they can be easily functionalized with any biomolecule, such as an enzyme, due to the adsorption properties of chitosan.^29^ In this work, the micromotors were modified with ATChE to further illustrate the use of micromotors in inhibition-based assays. For more details on micromotor synthesis, please see the Experimental Section.

Critical variables in micromotor synthesis were judiciously optimized to get the best propulsion and analytical performance. First, the concentration of the PB precursors was optimized. Speed of the micromotors increased along with the concentration of the precursors up to 0.05 M (from 20 ± 7 to 45 ± 12 μm/s, respectively) due to an increase in the amount of PB as a catalytic material. At higher concentrations, a dramatic decrease in the speed to 10 ± 5 μm/s is produced (0.2 M precursors), probably due to the increase in the kinetics of formation of Prussian Green, which also implies incomplete and inadequate encapsulation on the chitosan structure during the synthesis.^26^ The second variable optimized was the effect of the addition of glutaraldehyde as a cross-linking and reduction agent during synthesis.

Figure 2B shows the speed of cross-linked and un-cross-linked PB/chitosan micromotors at 3, 5, and 10% H_2_O_2_ levels. As can be seen, the micromotors synthesized using glutaraldehyde as the cross-linking agent show better performance as they were able to propel themselves at lower fuel concentrations (3%) at higher speeds. This is due to an increase in micromotor rigidity, which in turn improves the stability of PB encapsulation and will be very beneficial for future enzyme functionalization as well. Indeed, the SEM and EDX images of Figure 2C illustrate the defined tubular morphology of the micromotors with a uniform distribution of C (from chitosan) and Fe (from PB). The merged image illustrates that the C signal arises only from the chitosan layer and not from the inner PB. Under the optimized conditions (0.05 M PB and using glutaraldehyde as the cross-linker), the reproducibility of micromotor synthesis, in terms of micromotor speed, is excellent (see Figure S1, A), being stable around 1 week (Figure S1, B). Such capacity for rapid autodegradation will be beneficial for future *in vivo* biosensing and targeted drug delivery applications. In this sense, the biocompatibility of the micromotors was assayed by cell viability tests using the Caco-2 cell line with a colorimetric 3-(4,5-dimethylthiazol-2-yl)-2,5-diphenyltetrazolium bromide (MTT) assay. As shown in Figure S1C, the control experiments using sodium dodecyl sulfate (SDS) exhibit high toxicity, with cell viabilities of only 20% and 8%. The sodium cholate (NaCh) surfactant was almost nontoxic, with 86% cell viability. Almost 100% viability was obtained using 100.000 micromotors/mL and 1.5% NaCh, reflecting the high biocompatibility of our micromotors with living cells. Please note here that the aim of this article is to explore the (bio)-sensing capabilities of the PB micromotors rather than apply them to biomedical applications. To explore other applications, magnetic or other types of propulsion can be used, which do not require a peroxide or a surfactant. Furthermore, as it is the first time that PB is used as a catalyst for tubular micromotors, different surfactants were evaluated for their propulsion. As can be seen in Figure S2, the PB/chitosan micromotors do not propel themselves in SDS and Triton X-100; meanwhile, they propel effectively in Tween-20 and NaCh. This phenomenon is complex and can be caused by the influence of the surfactant on adequate bubble generation/nucleation and ejection.^30^ PB/chitosan micromotors indeed propel themselves better when NaCh is used as the surfactant, and it is shown to be a biocompatible surfactant in the MTT assay; all enzymatic colorimetric assays were performed using this surfactant. Also, as an iron composition material, PB can have magnetic properties. The magnetic susceptibility to the PB/chitosan micromotors was 1.1 × 10^–6^, demonstrating the paramagnetic behavior of our system. PB/chitosan can be magnetized under an external magnetic field, and it will lose the magnetization on removing the field. This magnetic property is very useful for simplifying separation steps during functionalization and sample analysis. As can be seen in Figure 2E, the micromotors get attracted by the magnetic block.

### Characterization of the Enzyme Mimetic Behavior of PB/Chitosan Micromotors

PB is a well-known catalyst for hydrogen peroxide decomposition, and it has been used as an “artificial” inorganic peroxidase and/or catalase for biosensor development. The enzyme-like activities of PB can be modulated as a function of the pH. Iron atoms present in the PB are responsible for the peroxidase- and catalase-like enzymatic activities. Also, it should be considered that the standard redox potentials of the pairs O_2_/H_2_O_2_ and H_2_O_2_/H_2_O are 0.7 and 1.8 V, respectively. At pH 4, the iron atoms present in the PB possess strong oxidation characteristics and are thus responsible for the peroxidase-like activity, promoting the decomposition of H_2_O_2_ into OH^–^ ions and OH^•^ radicals in a Fenton-like process.^3^ At pH 8, PB possesses catalase-like activity associated with the two electron-transfer channels, Fe^3+/2+^ or Fe(CN)_6_^3–/4–^.^25^ Indeed, the redox potential of H_2_O_2_/O_2_ is very low, and H_2_O_2_ is easily oxidized into O_2_ in a catalase-like mechanism.^3^

**Figure 3:**
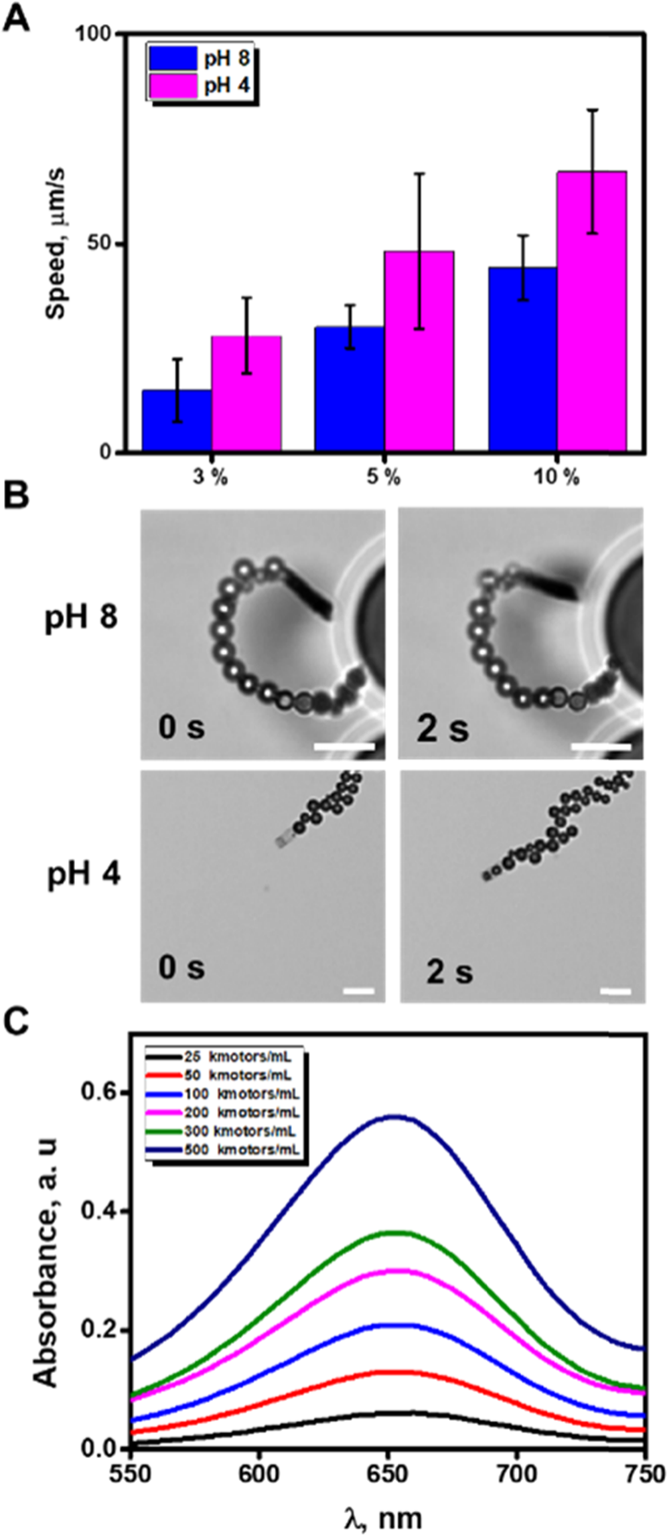
Catalase- and peroxidase-like enzymatic behavior of PB/chitosan micromotors. (A) Influence of pH on micromotor propulsion. (B) Time-lapse images (taken from Video S2) of the micromotor propulsion at different pH values. (C) UV–vis spectra corresponding to the oxidation of TMB in the presence of increasing numbers of micromotors. Conditions, 10% H_2_O_2_, 0.05 mg/L TMB, 1.5% sodium cholate, pH 4. Scale bar is 25 μm.

As such, first, we evaluated the enzymatic behavior of the PB/chitosan micromotors for propulsion function (see the concept in Figure 1) towards hydrogen peroxide decomposition at pH = 4 (peroxidase-like) and at pH = 8 (catalase-like). As can be seen in Figure 3A, the micromotors exhibit a higher yield of H_2_O_2_ decomposition at pH 4.0 than at pH 8.0, as reflected in the relatively higher speeds (1.5-fold) at the same fuel concentration level. Interestingly, at pH 4, PB is consumed during H_2_O_2_ decomposition (see Figure 3B and related Supporting information videos). Even the micromotors can get totally depleted from the catalyst material, resulting in an empty hydrogel tubular structure. The local production of OH^–^ in the vicinity of PB in a peroxidase-like mechanism leads to the release of soluble ferrocyanide and Fe^2+^, accelerating the decomposition and dissolution of the PB.^31^ Such results further support our hypothesis on the peroxidase-like behavior of our micromotors at acidic pH. At pH 8, micromotors propel autonomously and no apparent dissolution of the PB layer is observed, with unlimited navigation until H_2_O_2_ is depleted. Indeed, Figure S3 shows the speeds of the navigation of PB/chitosan micromotors at pH 8 over different periods. We track the micromotors, which move at overall speeds of 65 ± 15 μm/s. Also, to check the prolonged navigation, we kept the micromotors moving in an Eppendorf to prevent the solution from drying. After 30 min, a sample aliquot was placed in the microscope, and the speed of the micromotor was kept constant. In this case, as previously mentioned, the PB displays a catalase-like behavior, decomposing the H_2_O_2_ into O_2_. In this case, the absence of OH^–^ ions results in the stability of the PB structure, being an additional probe of our hypothesis.^3^

The slight speed observed at pH 4 compared to that at pH 8 can be associated with the dissolution of the PB layer at acidic pH. At this condition, the PB layer is gradually consumed, as such the weight of the micromotor is lower and thus higher speeds over time are reached.

Then, we evaluated the oxidation process of TMB mediated by PB/chitosan micromotors for colorimetric-based biosensing (see the concept in Figures 1 and 3C). To this end, we prepared TMB_red_ colorless solutions and mixed them with increasing numbers of micromotors. The TMB_ox_ solutions’ blue color and the absorbance at 650 nm increased along with the number of micromotors in the presence of 10% H_2_O_2_.

Yet, colorimetric assays using the micromotor technology present the drawback of bubble formation, which can interfere with colorimetric detection. Such a limitation can be avoided by exploiting the magnetic properties of the PB/chitosan micromotors to trap and remove them from the solution after color development. Yet, some microbubbles still remain in solution, which increase the background during colorimetric measurements. To minimize such an effect, prior to real assays, we optimized the amount of micromotors used by evaluation of the signal/background relation. Such a relation is close to 4 in the range of 25 000–1 00 000 micromotors/mL and decreases to a value of 3.5 for a higher concentration of micromotors (>1 00 000 micromotors/mL). As such, 1 00 000 micromotors/mL was selected as optimal due to the bigger signal and signal/background obtained.

### Neostigmine Determination Using PB/Chitosan Micromotors

**Figure 4:**
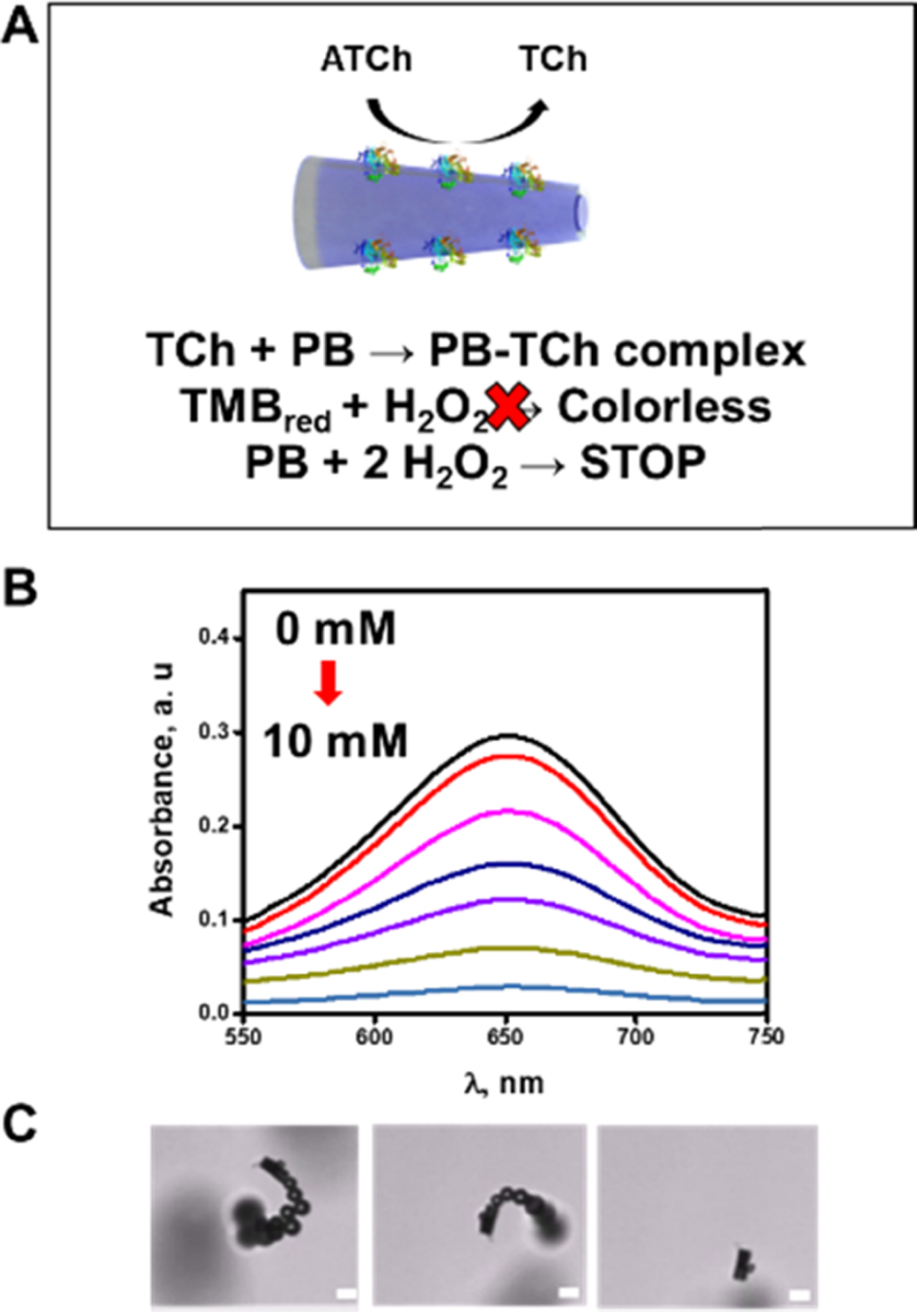
Principle of PB/chitosan/ATChE micromotor inhibition in the presence of TCh: (A) schematic of the process, (B) UV–vis spectra of TMB at different concentrations of TCh (0, 0.2, 1, 3, 5, 8, 10 mM), and (C) time-lapse images of a micromotor (taken from Video S3) over 0, 5, and 30 s navigation in solutions containing 10 nM ATCh. Assay conditions: 1 00 000 micromotors/mL, 10% H_2_O_2_, 1.5% NaCh, 0.2 mg/mL TMB, incubation time 5 min with ATCh and 1 min with TMB/H_2_O_2_. Scale bar is 10 μm, pH = 8.

As was described previously, neostigmine determination was carried out following an inhibition-based assay (see Figure 1 and reactions 1 and 2). To this end, PB/chitosan micromotors were functionalized with ATChE. First, we optimized the ATChE immobilization and substrate (ATCh) concentration. The principle of detection relies on the inhibition of the activity of PB.^32^ As can be seen in Figure 4A, at pH 8, ATChE catalyzes the decomposition of acetylthiocholine (ATCh) into acetate and thiocholine (TCh). The generated TCh can interact with PB between thiol groups and iron centers, which hampers H_2_O_2_ decomposition (PB peroxidase activity is inhibited). As a result, TMB_red_ is not oxidized, and the solution remains colorless. The mechanism was further corroborated by the complete stop of the micromotor motion in the presence of high concentrations of ATCh due to poisoning of the catalyst, avoiding peroxide decomposition.

Prior to assaying the neostigmine detection and evaluating the analytical performance, micromotor functionalization was optimized (see Figure S4) by evaluating different enzyme concentrations, incubation times, and temperatures. To this end, the PB inhibition signal was monitored using TMB as the colorimetric mediator. An increase of PB inhibition produces a decrease of TMB_ox_ (blue color) in the solution (see reactions 1 and Figure 4A). The increase of enzyme concentration produced an increment of PB inhibition due to the greater amount of TCh produced, reaching the highest inhibition yields at 200 U/mL (Figure S4A). An increase in the incubation time also improves the enzyme attachment to the micromotor surface, reaching the maximum inhibition after 4 h of incubation, followed by a slight loss of inhibition capacity probably due to saturation (Figure S3C). The incubation temperature was studied using three ATChE concentrations. The optimal temperature was found to be 37 °C. Thus, an incubation with 200 U/mL for 4 h at 37 °C was chosen as the optimal condition for chitosan/PB micromotor functionalization.

Next, the ATCh enzyme substrate concentration was optimized by evaluating the analytical performance of the optimized PB/chitosan/ATChE micromotors. Figure 4B shows the UV–vis spectra of different ATCh concentration solutions in ultrapure water. As can be seen, PB inhibition is observed from 2.0 to 10.0 mM ATCh. Such an inhibition is also reflected by the stop in the micromotor motion at increasing concentrations of ATCh (see the time-lapse images in Figure 4C). Thus, we chose a concentration of 5 mM as the optimal substrate concentration since it produced an almost complete PB inhibition and also because it can easily turn on the PB activity again by the addition of an ATChE poisoning agent as a nerve agent or a pesticide or, as in our proof-of-concept application, neostigmine.

**Figure 5:**
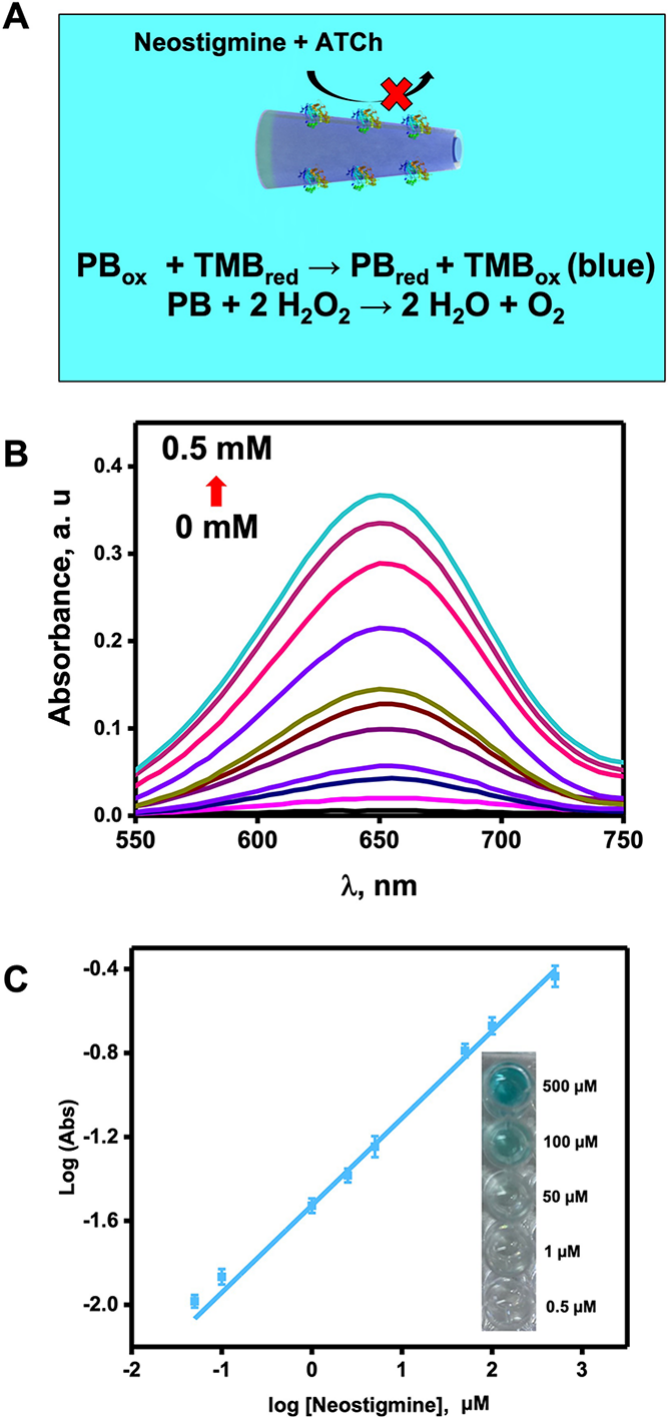
PB/chitosan/ATChE micromotors for neostigmine determination: (a) schematic of the principle for detection; (b) UV–vis spectra of TMB at different concentrations of neostigmine; and (c) corresponding calibration plot. Inset shows the color of the solutions at different concentrations of neostigmine (bottom). Assay conditions: 1 00 000 micromotors/mL, 10% H_2_O_2_, 1.5% NaCh, 0.2 mg/mL TMB, incubation for 10 min with neostigmine at 37 °C, 5 min with 5 mM ATCh at 37 °C, and 1 min with TMB/H_2_O_2_ at room temperature. pH = 4.

Once the bioassay conditions were optimized, we employed the PB/chitosan/ATChE micromotor for neostigmine determination as the ATChE inhibitor. The assay reactions are illustrated in Figure 5A and reaction 2. The analytical determination of neostigmine is carried out in two steps: (i) the inhibition of ATChE by neostigmine at pH 8 and (ii) the colorimetric detection toward the oxidation of TMB_red_ to TMB_ox_ at pH 4, without compromising the propulsion of the micromotors at such pH (for more details, see the captions of Figures 4 and 5).

The assays were performed by mixing the enzyme-modified micromotors, ATCh, TMB_red_, peroxide, and surfactant with solutions containing increasing concentrations of neostigmine. Such a compound is a reversible ATChE inhibitor, which can deactivate the enzyme and thus hamper the conversion of ATCh into TCh. Thus, the poisoning of the PB is prevented, acting as an active peroxidase mimic and promoting the generation of blue TMB_ox_. Thus, a higher concentration of neostigmine will produce a higher colorimetric signal. Figure 5B,C shows the UV–vis spectra of different solutions containing increasing concentrations of neostigmine along with the corresponding calibration plot in ultrapure water. As can be seen, calibration curves exhibit a linear correlation after logarithmic treatment of absorbance signals and neostigmine concentration (*r* = 0.990). An LOD and LOQ of 0.080 and 0.30 μM, respectively, were obtained for neostigmine with a linear range up to 500 μM. Moreover, all assays require less than 20 min in total. Also, as can be seen on the right of Figure 5C, naked-eye detection of neostigmine in solutions with a concentration above 50 μM can easily be achieved without any additional instrumentation.

Table S1 shows a comparison of the analytical performance of our method with other neostigmine-based detection approaches using PB and other enzymes like nanomaterials. For comparison, we process the data to obtain inhibitor concentrations needed for 50% inhibition of enzyme activity (IC_50_) (for further details and calculation details, see Figure S5). Please note that our method displays a higher IC_50_ (24 μM), which means that more amount of neostigmine is needed to inhibit 50% of the enzyme activity, indicating higher LODs. Yet, our linear range is the broadest of all of the methods found, which can be beneficial in future sensing avoiding sample dilution. In addition, the estimated lethal dose for neostigmine is >0.45 μM for a child for results obtained in mice,^33^ and the LOQ of our method is 0.30 μM; thus, it can be applied for the reliable determination of such a compound at relevant concentrations. Please note that the PB micromotor-based approach requires only 20 min for the overall (bio)-assay (15 min for incubation + 5 min for detection), where in the latter just 1 min was required for the color change due to the enhanced micromotor mixing, which allows for fast detection in microvolumes of the sample. It must also be considered that the comparison of our work with the bibliographic works is not easy because in the works listed in Table S1, the inhibition of the free enzyme in solution, which is the analyte, is studied. However, in our work, the enzyme is immobilized in the micromotor, in which ATChE inhibition is elegantly modulated on board the micromotor through the concentration of neostigmine, which is our target analyte.

Neostigmine intoxication can be accidental, but it can be used as a poison for homicidal purposes.^34^ To further demonstrate future applicability of the micromotors in this field, we evaluate the feasibility of the method in commonly consumed beverages. Samples without previous pretreatment were spiked with different concentrations of neostigmine, and recovery studies were performed. Table S2 summarizes neostigmine recovery in different samples. As can be seen, quantitative and reproducible recoveries were obtained in all samples and concentration levels, which show the quantitative capabilities of the micromotors to perform neostigmine determination in real domains. Moreover, the analytical approach described in this work can be used as a forensic kit, where the functionalized micromotors and ATCh can be added to the sample, incubated for 15 min “as a prepared reagent”. Then, TMB and hydrogen peroxide can be added for colorimetric detection in only 5 min at room temperature. This can be used as a screening tool for nerve agents for in situ analysis in crime scenes in a total 20 min assay.

## Conclusions

We have demonstrated the potential of PB/chitosan micromotors as artificial mimic enzymes for (bio)-sensing applications with excellent analytical performance.

The new approach integrates and exploits the PB mimic enzyme activities and magnetic properties for propulsion and detection operations for *on-the-fly* biosensing purposes. Hydrogel has become a key component in PB-based micromotor building as a supporting skeleton of these unique multifunction micromotors, which open new avenues in micromotor synthesis for a plethora of future analytical and even biomedical applications. Indeed, the chitosan structure allows its functionalization with other enzymes or bioreceptors, wherein the PB layer can be tailored for propulsion with metabolites such as urea or glucose in the presence of the corresponding enzyme, holding considerable potential for future applications only limited by our imagination.

In short, PB/chitosan/ATChE micromotors have shown exceptional analytical capabilities for neostigmine determination. A fast colorimetric assay (20 min) on microliter sample volumes without pretreatment was successfully achieved, and as such, this new approach holds considerable potential for in situ forensic analysis. In the light of these results, micromotor-based tests to be used in the crime scene are also envisioned.

## Supporting Information Available

Micromotor synthesis reproducibility in terms of speed; surfactant effect on micromotor propulsion at different fuel concentrations; speeds of the prolonged navigation of PB/chitosan micromotors; optimization of micromotor functionalization with the enzymeacetylcholinesterase; inhibition efficiency plot for neostigmine; comparison of the analytical performance of the method for neostigmine determination with colorimetric enzyme-like material-based methods; neostigmine determination in popular consumed beverages (PDF)
Prussian Blue/chitosan micromotor propulsion in different surfactants (AVI)
Prussian Blue/chitosan micromotor propulsion at different pH values (AVI)
Prussian Blue/chitosan micromotor navigation in ATCh solution (AVI)

## Supplementary Material

ac1c05173_si_001.pdf

ac1c05173_si_002.avi

ac1c05173_si_003.avi

ac1c05173_si_004.avi
