# Supplementary material for: Prussian Blue/Chitosan Micromotors with Intrinsic Enzyme-like Activity for (bio)-Sensing Assays
Source: Anal Chem. 2022 Apr 1;94(14):5575–82. doi: 10.1021/acs.analchem.1c05173 (PMC9008696; doi:10.1021/acs.analchem.1c05173)
Supplement: Supplementary file 1 — ac1c05173_si_001.pdf [file ac1c05173_si_001.pdf]

Prussian-blue/chitosan micromotors with intrinsic enzyme-like activity for (bio)-sensing assays

Roberto María-Hormigos,[†] Águeda Molinero-Fernández,[†] Miguel Ángel López,[†‡] Beatriz Jurado-Sánchez,[†‡] and Alberto Escarpa[†‡*]*

†Department of Analytical Chemistry, Physical Chemistry and Chemical Engineering, University of Alcala, Alcala de Henares E-28871, Madrid, Spain. E-mail: beatriz.jurado@uah.es, alberto.escarpa@uah.es (Tel: +34 91 8854995)

‡Chemical Research Institute "Andrés M. del Río", University of Alcala, Alcala de Henares E-28871, Madrid, Spain

Supporting figures

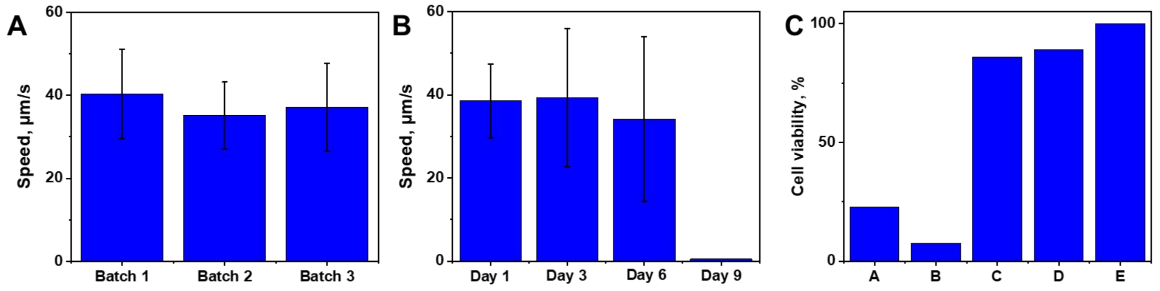

Figure S1. A) Micromotors synthesis reproducibility in terms of speed in 10 % of H_2O_2 and 1.5 % NaCh solutions. B) Micromotors stability along different days after storage in phosphate buffer 0.1 M pH 8.0 at 4 °C. C) Caco-2 cell viability obtain by MTT assay using hydrogen peroxide 10 % (A), SDS 1.5 % (B), NaCh 1.5 % (C), 1000000 chitosan/PB micromotors and NaCh 1.5 % (D) and 100000 chitosan/PB micromotors and NaCh 1.5 % (E).

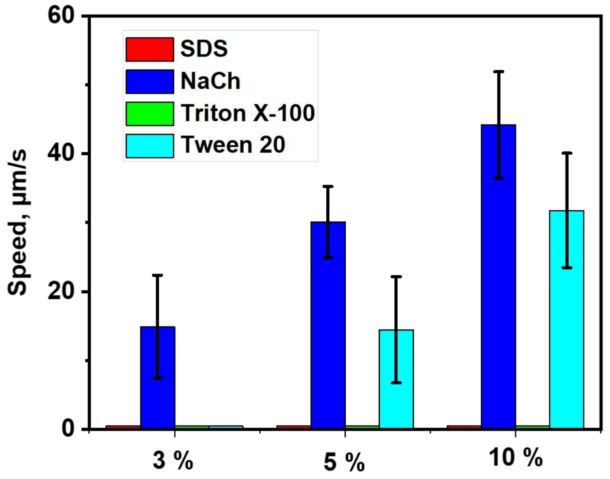

Figure S2. Surfactant effect on micromotors propulsion at different fuel concentrations. SDS, NaCh, Triton X-100 and Tween 20 concentration, 1.5 %.

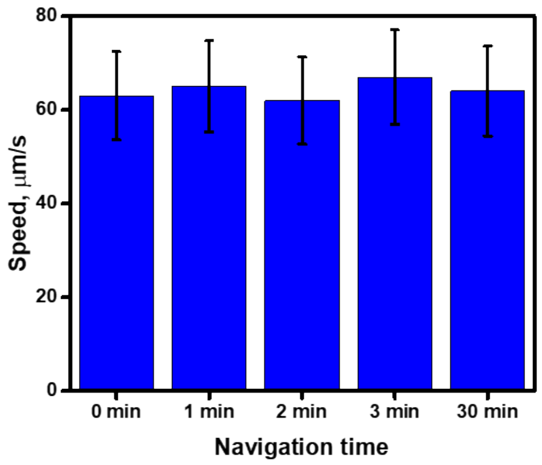

Figure S3. Speeds of the prolonged navigation of PB/chitosan micromotors. Conditions, 10 % H_2O_2, 0.05 mg/L TMB, 1.5 % sodium cholate, pH=8. Scale bars, 10 μm.

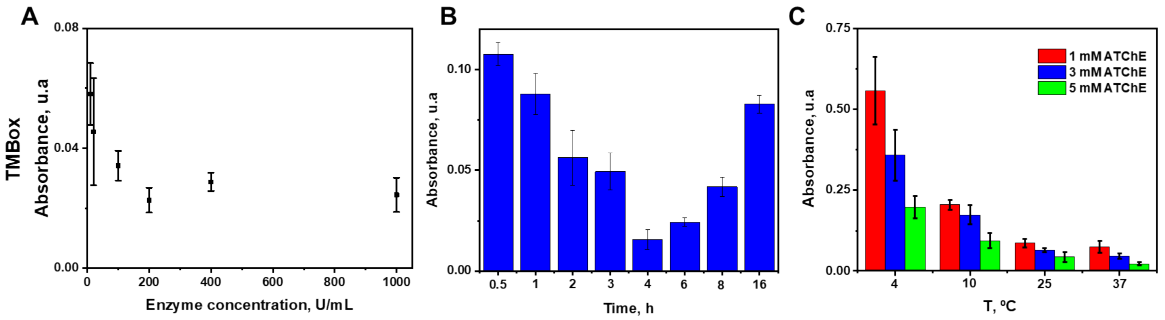

Figure S4. Optimization of micromotors functionalization with the enzyme acetylcholinesterase. A) Enzyme concentration. B) Incubation time. C) Incubation temperature. General assay conditions: 100000 micromotors/mL, 10 % H_2O_2, 1.5 % NaCh, 10 mM ATCh, incubation time 5 min with ATCh and 1 min with TMB/H_2O_2.

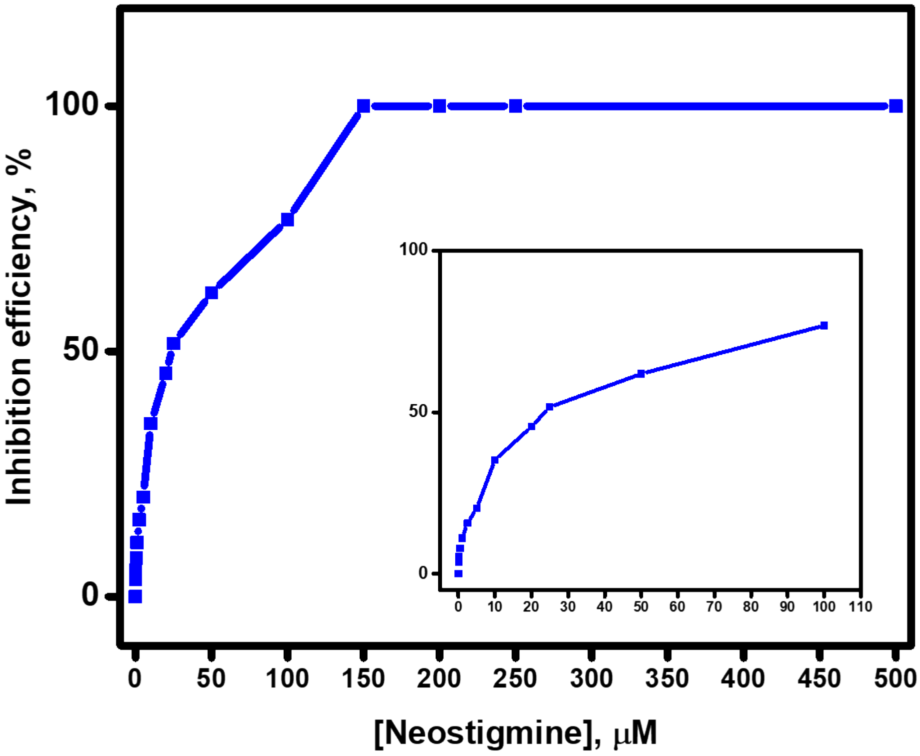

Figure S5. Inhibition efficiency plot for neostigmine. IC_{50} (%)= [(A_t–A)/(A_0–A)], where A and At are the absorbance values of the TMB solution in the absence and the presence of different concentration of neostigmine, respectively; and A_o is the absorbance in the absence of AChE.

Table S1. Comparison of the analytical performance of our method for neostigmine determination with colorimetric enzyme-like materials-based methods. All method uses TMB as colorimetric probe.

Material	Concentration inhibition, μM	IC_{50}, μM	Assay time (min)	Ref
PB nanocubes	1	0.010	55	[1]
Co_3O_4/MO_3 on ZIF-67	1	0.010	35	[2]
Black phosphorous quantum dots	60	0.09	80	[3]
Ag (I)	0.01	0.001	40	[4]
MnO_2 nanosheets	0.5	0.003	45	[5]
PB/chitosan micromotors	0.3-500[a]	24	20[b]	This work

[a]Inhibition concentration linear range.[b]15 minutes incubation time+ 5 minutes for the detection, with just 1 min required for the color change

Table S2. Neostigmine determination in popular consumed beverages.

Sample	Spiked concentration, μM	Measured concentration, μM	Recovery %
Tap water	0.5	0.48 ± 0.06	96 ± 13
	25	27 ± 2	108 ± 10
	250	230 ± 15	92 ± 14
Chamomile	0.5	0.40 ± 0.05	80 ± 12

infusion	25	25 ± 2	99 ± 8
	250	241 ± 12	97 ± 1
Beer	0.5	0.40 ± 0.04	80 ± 4
	25	25 ± 2	100 ± 9
	250	243 ± 10	97 ± 6
Black tea	0.5	0.53 ± 0.07	99 ± 14
	25	23 ± 2	92 ± 8
	250	243 ± 11	97 ± 6
Coffee	0.5	0.51 ± 0.08	95 ± 18
	25	28 ± 4	89 ± 16
	250	222 ± 19	89 ± 9

[a] Values are expressed as Mean Values ± S.D (n=3)

References

(1) Ni, P.; Sun, Y.; Dai, H.; Lu, W.; Jiang, S.; Wang, Y.; Li, Z.; Li, Z. Prussian Blue Nanocubes Peroxidase Mimetic-Based Colorimetric Assay for Screening Acetylcholinesterase Activity and Its Inhibitor. *Sens. Actuat. B.* **2017**, *240*, 1314.

(2) Zhang, X.; Lu, Y.; Chen, Q.; Huang, Y. A Tunable Bifunctional Hollow Co_3O_4/MO_3 (M = Mo, W) Mixed-Metal Oxide Nanozyme for Sensing H_2O_2 and Screening Acetylcholinesterase Activity and Its Inhibitor. *J. Mater. Chem. B.* **2020**; *8*, 6459.

(3) Ren, L.; Li, H.; Liu, M.; Du, J. Light-Accelerating Oxidase-Mimicking Activity of Black Phosphorus Quantum Dots for Colorimetric Detection of Acetylcholinesterase Activity and Inhibitor Screening. *Analyst.* **2020**,145, 8022.

(4) Ni, P.; Sun, Y.; Dai, H.; Jiang, S.; Lu, W.; Wang, Y.; Li, Z.; Li, Z. Colorimetric Assay for Acetylcholinesterase and Inhibitor Screening Based on the Ag [I] ion–3,3′,5,5′-tetramethylbenzidine (TMB). *Sens. Actuat. B.* **2016,** *226*, 104.

(5) Sun, Y.; Tan, H.; Li, Y. A Colorimetric Assay for Acetylcholinesterase Activity and Inhibitor Screening Based on the Thiocholine–Induced Inhibition of the Oxidative Power of MnO_2 Nanosheets on 3,3′,5,5′–tetramethylbenzidine. *Microchim. Acta.* **2018**, *185*, 446.
